# Membranous Dysmenorrhœa

**Published:** 1875-09

**Authors:** 


MEMBRANOUS DYSMENORRHŒA.—A correspondent sends us the ℞., which we give below, for Falk's Antacrid Tincture, which was published before the war in the New Orleans *Medical News and Hospital Gazette*, by Dr. Fenner, and highly extolled by him as a remedy in dysmenorrhœa, and also for the sterility which so frequently attends upon that malady. Our correspondent, himself, has used the remedy for years, and with very uniform and happy results. We too have had a very happy experience in the use of the same prescription in the common forms of dysmenorrhœa, and even in true membranous dysmenorrhœa we have found the remedy useful in *mitigating the pains* attendant upon the epoch; but neither this nor any other remedy which has come to our notice has proved effective in controlling the expulsion of a *true dysmenorrhœal membrane,* in permanently curing the malady known as membranous dysmenorrhœa, or in inducing pregnancy in a married woman laboring under the disease. We hope, however, to do a good service to our younger readers by reproducing the formula of Dr. Fenner for

FALK'S ANTACRID TINCTURE.

| | |
|---|---|
| ℞.—Gum Guaiac, . . . . . . | ℥j. |
| Canada Balsam, . . . . . . | ℥j. |
| Oil Sassafras, . . . . . . | ʒij. |
| Corrosive Sublimate, . . . . . | ℈j. |
| Alcohol, . . . . . . . . | ℥viii. |

Dissolve the guaiac and balsam in one-half the spirits and the sublimate in the other. Let the former digest for a week; then pour off the clear liquor, mix with the sublimate and add the oil.

Dr. Fenner directs: "I usually direct the patient to begin a day or two before the expected period, and take *twenty-five* drops in an infusion of sage or sweetened water, night and morning, until the discharge is freely established; then cease until the next period. In obstinate and severe cases, the medicine should be commenced a week or ten days before the period; and if the pain appears, the medicine should be taken every four or six hours until relieved. (It should be borne in mind that the dose of twenty-five drops represents nearly, or quite, *one-twelfth* grain of corrosive sublimate.—Ed.) The pain usually disappears as soon as the discharge becomes free; but in most cases the discharge comes on without pain after taking a few doses. I have known immediate relief to be given by a single dose, taken in the paroxysm; but I have seen cases in which the pain was excruciating, causing shrieks and even violent convulsions. In such I have had to resort to a more prompt and efficient anæsthetic, as the inhalation of chloroform, or the following, which I have often known to act like a charm:

℞.—Spirit camphor, . . . . . . ℥iij.

Chloroform, . . . . . . . ℥ij.

Tinct. Opium, . . . . . . ℥j. M.

"Sig. A teaspoonful, in sweetened water, once an hour until relieved.

"In violent hysterical spasms there is nothing comparable to the inhalation of chloroform. In the treatment of dysmenorrhœa it is important to obviate costiveness by the use of aloetic pills. When dysmenorrhœa is relieved by this treatment, conception almost invariably soon occurs in married women."

---

We learn that Dr. James H. Pooley, of Yonkers, N. Y., favorably known to our readers by his occasional contributions to our columns, has accepted the Professorship of Principles and Practice of Surgery in the Starling Medical College at Columbus, Ohio.

